# Temporal diversification of Central American cichlids

**DOI:** 10.1186/1471-2148-10-279

**Published:** 2010-09-14

**Authors:** C Darrin Hulsey, Phillip R Hollingsworth, James A Fordyce

**Affiliations:** 1 Department of Ecology and Evolutionary Biology, University of Tennessee, Knoxville, TN, USA

## Abstract

**Background:**

Cichlid fishes are classic examples of adaptive radiation because of their putative tendency to explosively diversify after invading novel environments. To examine whether ecological opportunity increased diversification (speciation minus extinction) early in a species-rich cichlid radiation, we determined if Heroine cichlids experienced a burst of diversification following their invasion of Central America.

**Results:**

We first reconstructed the Heroine phylogeny and determined the basal node to use as the root of Central American Heroine diversification. We then examined the influence of incomplete taxon sampling on this group's diversification patterns. First, we added missing species randomly to the phylogeny and assessed deviations from a constant rate of lineage accumulation. Using a range of species numbers, we failed to recover significant deviations from a pure-birth process and found little support for an early burst of diversification. Then, we examined patterns of lineage accumulation as nodes were increasingly truncated. We assumed that as we removed more recently diverged lineages that sampling would become more complete thereby increasing the power to detect deviations from a pure-birth model. However, truncation of nodes provided even less support for an early burst of diversification.

**Conclusions:**

Contrary to expectations, our analyses suggest Heroine cichlids did not undergo a burst of diversification when they invaded from South America. Throughout their history in Central America, Heroine cichlids appear to have diversified at a constant rate.

## Background

Patterns and rates of speciation vary tremendously among clades. Vertebrate groups such as coelecanths or bowfin fishes that were once species rich, underwent bouts of extinction, and now are represented by few extant species [1]. Alternatively, diversification (speciation minus extinction) in other clades such as cichlid fishes can be explosive. In several instances, cichlids have diversified incredibly rapidly resulting in species assemblages that dominate many freshwater habitats [2-4]. Both intrinsic and extrinsic forces could underlie the tendency of cichlids to rapidly diversify. Intrinsic factors such as innovations in jaw structure [5,6] and reproductive phenotypes like mouth brooding [7] have been suggested to drive rapid diversification in these fishes. However, in the largest of the East African Rift lakes, extrinsic factors such as the ecological release these cichlids encountered upon colonizing Lakes Tanganyika, Victoria, and Malawi could have fueled their bursts of unparalleled diversity [3,8]. The absence of predators or competitors in these three massive, relatively old lakes could have been the key to the rapid filling of niches with the hundreds of endemic cichlid species that now occur sympatrically in each lake. However, it is generally unclear how important the invasion of novel habitats is to cichlid diversification [9] and if cichlids presented with novel ecological circumstances frequently experience a burst and subsequent slow-down in cladogenesis. To examine the hypothesis that novel ecological opportunity facilitates an increase in cichlid diversification rate, we determined whether Heroine cichlids underwent a burst of diversification following their invasion of Central America.

Central American (CA) Heroine cichlids are thought to represent an adaptive radiation that like many other cichlid radiations could have undergone a burst of diversification early in their history. Based on one taxonomic database [10] and recent species descriptions [11-13] there are 122 named species in this clade with 114 of these inhabiting the region north of the Panamanian Isthmus. These fishes are also extremely trophically diverse and exhibit an incredible array of putatively adaptive jaw morphologies [14-18]. The Heroines are nested phylogenetically within a more evolutionarily disparate group of cichlids [19] that has occupied South America since at least the Eocene, 40-55 million years ago [20]. The diversity in CA Heroines has been attributed in part to the paucity of Central American representatives of groups like catfish and characiform fishes that dominate South American freshwater ecosystems [21,22]. Additionally, the few other cichlid lineages that occur north of the Panamanian Isthmus, *Geophagus *and *Acarichthys*, have only a few Central American species and likely did not colonize Central America until the emergence of the Isthmus of Panama, 3 million years ago [23]. Because of the lack of competing fish lineages, Central America likely offered a huge opportunity for adaptive divergence when Heroine cichlids invaded this region from South America. However, it is unclear if the adaptive diversity and species richness of the CA Heroines is largely a result of an early burst of explosive speciation as is predicted under many scenarios of adaptive radiation [24,25].

There are several microevolutionary studies that suggest that the diversification of CA Heroines continues to occur rapidly. This radiation contains some of the best-documented cases of intraspecific trophic polymorphism in vertebrates [26,27]. For example, species such as *Herichthys minckleyi *and *Amphilophus citrenellus *exhibit polymorphic jaw morphologies that allow individuals within the same populations to exploit highly different prey types [27,28]. Some of the most thoroughly examined cases of sympatric speciation also occur in this radiation [29-31]. These cases of on-going divergence in the Heroine cichlid radiation suggest that speciation in these fish is strongly influenced by ecological opportunity. Also, these instances of recent divergence could be used to argue diversification in the CA Heroine radiation has not slowed since the Heroine invasion of Central America.

Many large radiations like the CA Heroines exhibit clear patterns of slowdown in diversification towards the present [32,33]. Evaluating the patterns of diversification during a clade's history requires the ability to assess the temporal patterns of cladogenesis. Recent advances in the ability to estimate phylogenies and branch lengths have made evaluating temporal patterns of speciation feasible [34-38]. Lineage through time (LTT) studies also depend on time-calibrated phylogenies [34] and a determination of which ancestral node is relevant to begin examining the effects of novel conditions on clade diversification. Fortunately, an increasingly robust view of the interrelationships of CA Heroine cichlids is coming to light. Also, because of the ability to reconstruct ancestral areas, such as inhabiting Central America or not, and the relatively clear colonization of CA Heroines from South America, determining which cichlid clades to include in a LTT analysis should be feasible. If Heroine cichlids underwent an explosive phase of lineage diversification when they colonized Central America, this pattern should be detectable.

When evaluating the temporal variation in diversification rate of an adaptive radiation like the CA Heroines it is critical to account for incomplete sampling of lineages [35]. Species in a molecular phylogeny can be incompletely sampled because of unavailability of DNA sequences for known lineages and/or taxonomic inaccuracies in the delineation of species diversity. Simulations can be used to correct for an incomplete sampling of lineages in a phylogeny via the creation of null distributions that generate random trees and subsequently prune lineages from phylogenies [35,37]. Both underestimation of the number of "true" lineages [39] and overestimation of the number of species can also lead to inaccurate estimation of species diversity [40,41]. This taxonomic inaccuracy likely influences the evaluation of recent LTT patterns most. To remedy this problem, many LTT studies have somewhat arbitrarily truncated the most recent 2 million years of the topology to reduce the influence of taxonomic inaccuracy on the estimation of recent diversification [42]. Pruning a range of times or nodes from a topology to assess how taxonomic inaccuracies influence deviations from null expectations would provide an even more robust method to account for taxonomic inaccuracies. If Heroine cichlids underwent an explosive phase of lineage diversification when they colonized Central America, this pattern should be detectable with analyses that account for uncertainty in the number of extant lineages.

We combined phylogenetics, ancestral state reconstruction, and simulations to assess the temporal patterns of CA Heroine diversification. We first reconstructed the phylogeny of CA Heroines and subsequently used likelihood reconstruction to determine the basal node from which to estimate patterns of diversification. Then, we used simulations and the truncation of nodes to examine Heroine deviations from a null distribution of diversification expected under a pure-birth, constant rate model. We combined these analyses to examine if patterns of Heroine diversification were consistent with rapid lineage accumulation coincident with their invasion of Central America.

## Results

### Phylogeny Reconstruction

**Figure 1:**
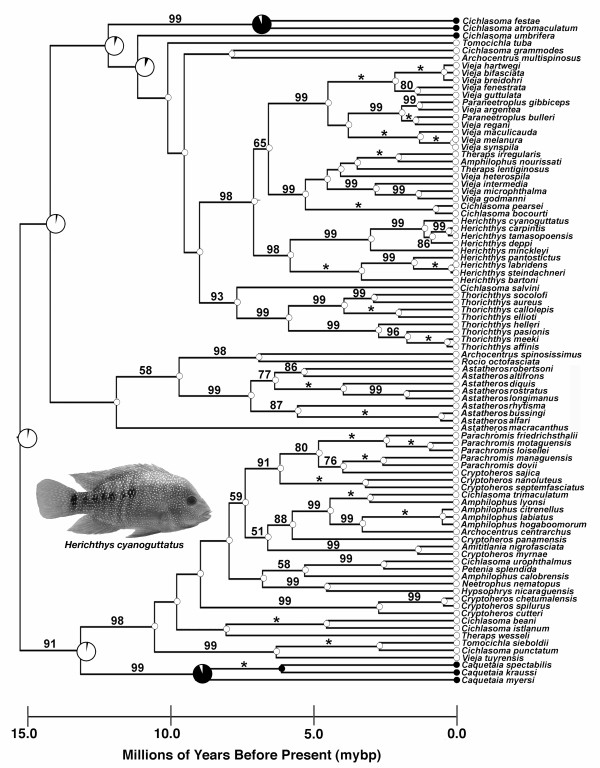
**Chronogram of CA Heroine diversification**. Likelihood ancestral areas are reconstructed at nodes as either Central American (white) or South American + Greater Antilles (black). The tips as well as many of the nodes in the phylogeny are only one color and therefore are reconstructed with virtually 100% probability as one state. The pie diagrams that exhibit both colors represent ambiguous likelihood reconstructions of the area at that node and are depicted at larger sizes. Posterior probabilities of nodes are shown on the branch preceding the nodes and are indicated with an * if there is 100% Bayesian support for the node. Other numbers behind the nodes represent the percentage that that node was recovered among topologies saved in the Bayesian analysis. Only support values above 50% are depicted. The node calibrated with divergence of 7.5 million years before present (mybp) around the Punta del Morro is shown with an **X**. A temporal scale running from 15.0 mybp to the recent is shown below the chronogram. An image of a typical Central American Heroine cichlid, *Herichthys cyanoguttatus*, is shown nested within the phylogeny.

The phylogenetic relationships recovered with the 17 new cytochrome *b *sequences (Table 1) analyzed in a Bayesian framework do not differ greatly from previous analyses using the cytochrome *b *gene [43,44]. The clade of three Greater Antillean Heroines and the South American *Australoheros*, that in some phylogenetic analyses [45-47] have been recovered as nested within the CA Heroines, fell phylogenetically just outside of the CA Heroine clade here (Figure 1). These groups would not be considered as primarily diversifying in Central America so their phylogenetic position inferred here was also convenient for our analyses. None of the previously sequenced species that we re-sequenced here to better estimate branch-lengths exhibited novel relationships.

**Table 1:** Species sequenced for cytochrome *b *for this study.

Species	Genbank	Collection Locality
*Archocentrus spinosissimus*	[Genbank: HM193439]	commercial
*Cichlasoma beani*	[Genbank: HM193451]	Río Tepic, Nayarit, MX
*Cryptoheros chetumalensis*	[Genbank: HM193442]	Río Hondo, Quintana Roo, MX
*Cryptoheros cutteri*	[Genbank: HM193448]	Río Lancetilla, HN
*Cryptoheros spilurus*	[Genbank: HM193449]	Río Gracias a Dios, GT
*Paraneetroplus gibbiceps*	[Genbank: HM193450]	Arroyo Cristal, Tabasco, MX
*Parachromis friedrichsthalii*	[Genbank: HM193440]	Orange Walk, BZ
*Parachromis managuensis*	[Genbank: HM193452]	Lago de Yojoa, HN
*Parachromis motaguensis*	[Genbank: HM193453]	Río Choluteca, HN
*Thorichthys affinis*	[Genbank: HM193438]	Río Hondo, Quintana Roo, MX
*Thorichthys aureus*	[Genbank: HM193446]	Río Amatillo, GT
*Thorichthys meeki*	[Genbank: HM193445]	Lago de Illusiones, Tabasco, MX
*Thorichthys pasionis*	[Genbank: HM193447]	Lago de Illusiones, Tabasco, MX
*Thorichthys socolofi*	[Genbank: HM193443]	Río Macuspana, Tabasco, MX
*Vieja bifasciata*	[Genbank: HM193444]	Río Tzendales, Chiapas, MX
*Vieja hartwegi*	[Genbank: HM193441]	Río Tzendales, Chiapas, MX
*Vieja maculicauda*	[Genbank: HM193454]	Río Lancetilla, HN

Genbank accession numbers are followed by the collection localities that include commercial sources, Guatemala (GT), Honduras (HN), and Mexico (MX). For geographic clarity, the states of collection sites in Mexico are provided.

For species that were not previously sequenced but fell within the 90 included CA Heroine species, we recovered several interesting relationships. *Thorichthys socolofi *was found to be sister to *T. aureus*, and *T. affinis *was recovered as sister to *T. meeki*. Interestingly, the species *Archocentrus spinosissimus *was placed as sister to *Rocio octofasciata *although the length of the branch separating these two species suggests they are highly divergent. *Parachromis friedrichstahlii *was recovered as sister to the clade containing *Parachromis motaguensis *and *Parachromis loiselli*. The two species *Cryptoheros cutteri *and *C. chetumalensis *that were recently taxonomically split out of *Cryptoheros spilurus *[12] formed a monophyletic group with *C. cutteri *being the most phylogenetically distinct.

### Chronogram

The root of the CA Heroine clade examined was estimated to be 16.2 million years before present (mybp), but there are several CA Heroine clades that appear to have diversified within a relatively recent timeframe (< 3 mybp). The *Amphilophus citrenellus + Am. labiatus + Am. hogaboomorum *clade forms a polytomy that likely includes a substantial number of species [31] that diverged very recently. The newly erected species *Cryptoheros chetumalensis *is also highly genetically similar to its sister *C. spilurus*. *Thorichthys meeki *and *T. affinis *as well as *Vieja melanura *and *V. synspila *are not genetically very distinct. Likewise, *Vieja hartwegi + V. bifasciata + V. breidohri *show very little sequence divergence. The sister clade to the polymorphic cichlid *Herichthys minckleyi *that includes *H. cyanoguttatus, H. deppi, H. carpintis*, and *H. tamasopoensis *also diverged within the last 3 million years.

### Ancestral Area Reconstruction

The likelihood reconstruction of ancestral areas indicates that there is greater than 95% probability that the ancestral lineage leading to the basal node in Figure 1 began diversifying in Central America. The estimated Mk1 rate parameter was 0.007 indicating a generally low rate of transition between Central America and either the Greater Antilles or South America. Because the three species of Greater Antilles cichlids fell outside of this clade it suggests they did not colonize this region by first occupying Central America. Our ancestral reconstruction also supports two instances of the CA Heroines re-colonizing and experiencing limited diversification in South America. The *Caquetaia *clade of three species as well as the clade composed of *Cichlasoma festae *+ *C. atromaculatum *+ *C. umbrifera *clade are both reconstructed here as re-colonizing South America. Retention of all of these species in the LTT analysis should not be problematic because analyses that pruned these species (results not shown) did not differ substantially from those we present. Also, these South American species represented relatively few species, their speciation times did not seem skewed in any particular direction, and there was some ambiguity (Figure 1) about where (*i.e*. South America vs. Central America) the speciation events at particular ancestral nodes took place.

### CA Heroine Lineages Through Time

**Figure 2:**
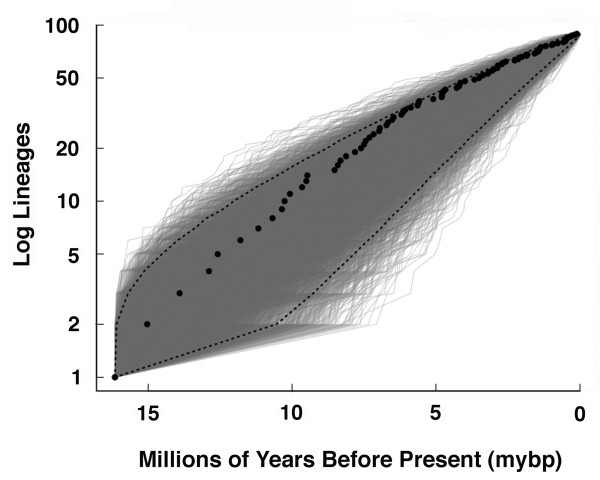
**The lineage-through-time (LTT) plot of CA Heroine diversification**. The black dots represent actual numbers of Heroine lineages estimated at a given time frame from the reconstructed phylogeny. The grey lines form an ellipse of simulated pure-birth phylogenies of 90 species used to compare to the CA Heroines. The dotted lines represent the 95% confidence interval of the number of lineages expected from the simulated pure-birth phylogenies. The CA Heroine LTT lies within the 95% null distribution expected for a radiation diversifying via a pure-birth process.

The lineage-through-time (LTT) plot provides no obvious excess of lineages early in the history of the Heroine cichlid radiation (Figure 2). The simulation of 10000 lineages using a pure-birth model to create a 95% CI for the expectation of diversification showed that during their entire diversification the accumulation of lineages in the CA Heroine clade fell within this null expectation. The only component of the Heroine radiation that might be skewed towards faster than expected diversification contains lineages originating within the last five million years. The virtual linear accumulation of species through time (Figure 2) is highly suggestive of a pure birth process governing diversification in CA Heroines.

**Figure 3:**
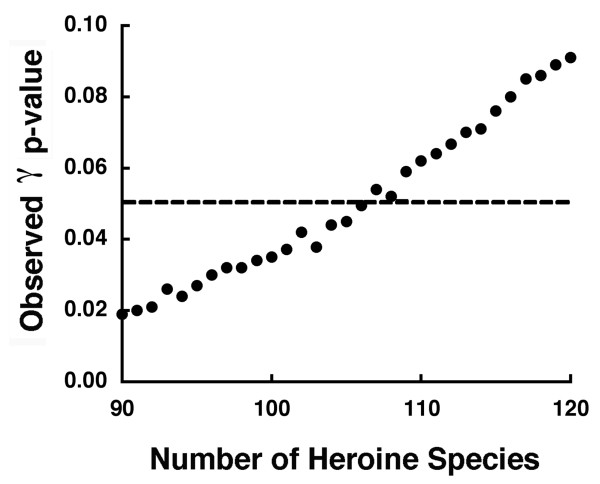
**Species number and γ statistic**. We used null distributions to test if γ exceeded the value of -1.645 (the critical value for α = 0.05) with a range of "true" total numbers of CA Heroine species in the clade. We examined γ with 90 species, the number of taxa sampled in the phylogeny, to 120 species. Increasing the number of species resulted in lower and lower probability of there being a burst early in the phylogeny. We therefore could not reject the hypothesis that CA Heroine cichlids exhibited a pure-birth model of constant lineage diversification if there are 122 species as current taxonomy suggests.

Based on the values of γ, the inference that there is a burst of diversification early in the clade of Heroine cichlids also depends upon how complete our taxon sampling of lineages is (Figure 3). If our phylogeny contains all of the species in Central America and all un-sampled lineages are merely synonyms of the 90 species currently sampled, then the chronogram of CA Heroines would exhibit a significant deviation from a pure-birth process. If this were true, CA Heroines might exhibit a slight burst of diversification early in the clade's history. However, this is unlikely, and if there are in fact 122 species of Heroine cichlids in this clade as current taxonomy suggests, this group does not show a burst of diversification early during its radiation.

**Figure 4:**
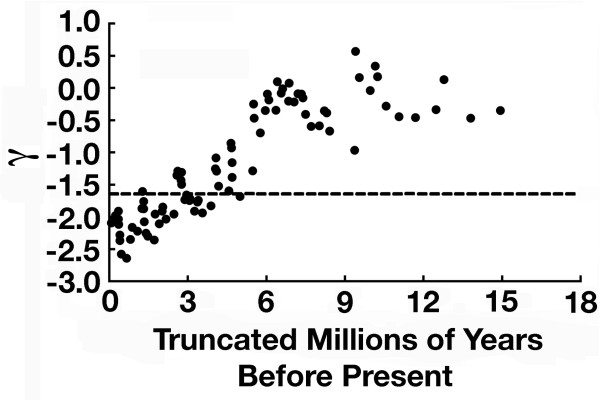
**Node truncation and stability of γ**. Nodes were truncated from the present to the root of the CA Heroine phylogeny in order to assess the stability of γ using our empirically estimated 90 species phylogeny. Because lineage sampling should become more complete towards the root of the phylogeny, truncation of nodes closer to the present should reduce the influence of incomplete lineage sampling on inferences made from γ. Node removal and γ recalculation is depicted temporally based on the last removed node's time-calibration from the CA Heroine chronogram. Importantly, once nodes from the present to 3.5 mybp are removed, γ is clearly not significant in our empirical phylogeny. Therefore, analyses of only the deeper nodes in the CA Heroine phylogeny cannot reject a pattern of pure-birth lineage diversification.

The truncation of nodes (Figure 4) indicates there is little support for a burst of diversification especially as one proceeds back into the CA Heroine phylogeny. Sampling of lineages should become more complete as one moves towards the root of the phylogeny, and therefore, the results of this truncation should test how robust our results are. If incomplete taxonomic sampling at the tips were skewing our inferences away from detection of a burst of diversification, we would expect that the γ values would become, or remain, more significant as nodes were truncated. However, we recovered the opposite. After nodes at an age of approximately 3.5 mybp were removed, the remaining γ values were clearly not above the critical threshold value of -1.645 that statistically marks the 95% expectation of a constant diversification rate. At 4.0 mybp and deeper in the phylogeny, the fact that γ did not exceed this critical value indicates there was not an early burst of diversification in CA Heroines. As nodes were truncated from our taxonomically incomplete chronogram, the γ values approached the expectation under a constant rate, pure-birth model of diversification. Both our simulations accounting for our incomplete phylogenetic sampling of the named Heroine species in Central America and pruning the topology back in time are concordant in suggesting there was no early burst of diversification in CA Heroine cichlids.

The expected rate of lineage accumulation under a constant-rate model of diversification cannot be rejected for the Heroine cichlids. Additionally, the model fitting approach failed to favor any of the rate variable models examined over a pure-birth model of lineage diversification. Regardless of the expected number of terminal taxa (90-122) in the clade, a pure-birth model (no extinction) could not be rejected for CA Heroine cichlids.

## Discussion and Conclusion

There was no dramatic burst of speciation as Heroine cichlids colonized Central America. The phylogenetic inclusion of several species further resolved the phylogeny of the entire clade, but did little to change the overall topology of the tree recovered in previous studies. The temporal estimates of the root node of Heroine cichlids as being close to 16 mybp is also consistent with most other molecular clock estimates derived from mitochondrial DNA markers for these fishes [44,48]. These studies, largely based on cytochrome *b *suggest an age of between 15 and 22 mybp for Heroine diversification. However, several recent studies indicate that Heroines and other cichlids groups are much older than previously thought [40,49,50]. These studies suggest that the reciprocally monophyletic diversification in the Neotropics and Africa is generally consistent with a Gondwanan split and divergence time estimates for each continental radiation of around 65-85 mybp. If this is true, Heroine cichlids could have begun diversifying in Central America almost 30 mybp [50]. Regardless, if our dates are close to realistic, it suggests that there are several clades in the CA Heroines that have likely diverged relatively recently (< 3 mybp). Nevertheless, our analyses do not provide substantial support for an increased rate of diversification at the base of the CA Heroine phylogeny.

This phylogenetic analysis is the most thoroughly sampled species-level analysis of Heroine cichlid relationships. However, it is unclear how incomplete the taxonomic sampling of the phylogeny is currently. Future studies of the alpha taxonomy of CA Heroines could substantially increase the number of named species in this group. There remain regions of Central America such as the flooded forests of the Moskitia in Honduras and Nicaragua where the diversity of freshwater cichlids has not been extensively documented [23]. Also, substantial numbers of CA Heroine lineages could be described by taxonomically splitting currently recognized species [12]. For instance, the species '*Cichlasoma*' *uropthalmus *that is represented in our phylogeny by a single lineage has 11 currently recognized species/subspecies [51]. If they form a recently evolved monophyletic group as is likely, the addition of these species to our phylogeny would further decrease support for a burst of diversification at the base of the CA Heroine radiation. The number of cichlid species in the crater lakes of Lake Nicaragua is also debated but the speciation times for these groups are clearly recent as well [31]. A complete tally of CA Heroines will undoubtedly recognize more lineages and generally increase the number of recent nodes towards the tips of the phylogeny rather than at the base of the radiation. Additional Heroine species descriptions will not skew diversification patterns toward detection of a burst of diversification at the base of this clade.

If there are in fact 122 species of Heroine cichlids in this clade, this group's diversification does not show a significant deviation from a pure-birth process (Figure 3). However, the inference that there could have been a burst of diversification in Heroine cichlids depends in part on the completeness of our taxon sampling. If our phylogeny contains all of the species in Central America and all un-sampled lineages are merely synonyms of the 90 species currently sampled, then the phylogeny of Central American cichlids would exhibit a significant deviation from a pure-birth process. However, because current taxonomy suggests there are 122 evolutionarily independent lineages, Heroine cichlids probably did not exhibit a burst of diversification concordant with their invasion of Central America. There is also no empirical support for a substantial amount of extinction (i.e. birth-death model) or temporal change (rate variable model) during the diversification of CA Heroine cichlids. The comparison of the LTT plot to null distributions and truncation of nodes provide little support for Heroine diversification deviating significantly from a pure-birth process. In fact, node truncation suggests that as one proceeds back in time, the historical pattern of CA Heroine diversification becomes less and less compatible with an early burst of diversification.

There remain a few caveats to this study. First, the use of a mitochondrial marker like cytochrome *b *could show saturation and thus underestimate the branch lengths connecting deeper nodes in the tree [52]. However, this would only bias analyses towards detection of a burst of speciation at the base of the radiation because of underestimation of older branch lengths. Hybridization could also mislead the inferences we are making with respect to CA Heroine phylogeny. However, hybridization is likely more important during the first few million years of diversification within clades of cichlids [53]. Therefore, the possibility of recent hybridization should not obscure our inferences of a lack of rapid lineage diversification in Heroine cichlid lineages that diverged around 16 mybp or even earlier. Analyses of more genes will undoubtedly provide an increasingly robust view of the phylogenetic relationships among this diverse clade. But, we have no reason to believe that the marker used here provides an exceptionally biased view of the diversification history of CA Heroines.

This study could call into question the general importance of ecological opportunity to cichlid diversification. However, one way that hypotheses aimed at elucidating the importance of ecological release to diversification could be refined is to more explicitly incorporate the history of these radiations into their particular environmental frameworks. There are several reasons the ecology of the African Great Lakes might better facilitate obvious bursts of diversification compared to other freshwater systems cichlids inhabit. Unlike rivers, the African Great Lakes contain archipelagos of small rocky islands [3] that favor rapid isolation and divergence. Additionally, the cichlid radiations in the African Great Lakes diversified in an exceptionally sympatric context. The radiation of CA Heroine cichlids likely involved a much more allopatric mode of diversification in the numerous river basins that dissect Central America. Lake Tanganyika is also likely well over 10 million years old [54] and could be as old as 20 million years [49]. Although Lake Victoria is less than a million years old [54] and Lake Malawi is likely close to 3 million years old [40,50], most of the lake and riverine habitats in Central America are young and geologically dynamic [55,56] as compared to the African Great Lakes. The extensive bathymetry of the African Great Lakes also create stratified light environments that appear to influence sexual selection and rapid reproductive isolation among lineages [53]. The exceptional number of herbivores and planktivores in the African Great Lakes [3,8] likewise could indicate there are ecological opportunities available in these habitats that are largely absent from other aquatic systems. Perhaps it is the particular types of ecological conditions and their stability over time that facilitated the explosive diversification of cichlid lineages inhabiting the East African Rift Lakes [9].

Many models and empirical studies suggest that adaptive radiations should be characterized by a burst of diversification early in their history [25]. We found no evidence that the CA Heroine cichlids exhibit a burst early in their history. However, we cannot exclude the possibility that CA Heroines are indeed undergoing rapid diversification and are still in the "early" stage of their radiation where it is impossible to detect a slowdown in diversification at this time because it has not yet occurred. If density dependence among members of a clade governs the rate of diversification [57], CA Heroines could also simply be far from the carrying capacity for the number of cichlid lineages that can co-exist. Currently, the maximum number of CA Heroine species that occur sympatrically is approximately 12 species [4]. This is far less than the 100 or more cichlid species that can occur sympatrically in some habitats in Lake Malawi [58]. The dynamic nature of the largely riverine and geologically active Central American landscape relative to the East African Great Lakes could have enforced a relatively slow fuse on the explosive diversification rate of CA Heroines.

Furthermore, although the rate of diversification in some East African Rift Lakes is clearly high [54,59-64], only diversification patterns in the oldest of the lakes, Lake Tanganyika, has been tested extensively in a LTT framework. Somewhat counter to what we find here for CA Heroines, diversification rate does appear to have slowed slightly towards the present in Lake Tanganyika. However, this slowdown was not as marked as one might expect and it is clear that the diversification rate is not especially rapid for the cichlids inhabiting this lake [64]. However, the diversification rates in Lake Victoria and Lake Malawi are unquestionably fairly rapid [64]. If CA Heroines are in fact only 16 million years old, diversification in this group of over 100 species has been rapid compared to many fish radiations [65,66]. However, if this clade is as old as 30 million years as some calibrations suggest [40,49,50], the diversification rate is likely to be more in line with many other fish groups. With multiple genes and multiple temporal calibrations, whether CA Heroines exhibit a relatively rapid diversification rate per million years could be tested. Additionally, ecological divergence in CA Heroines is substantial [4,15,17] and the rate of adaptive phenotypic and ecological divergence in this radiation could well be extraordinary. General processes, both intrinsic and extrinsic, that are responsible for generating diversity in adaptively diverging clades should come to light as radiations like the CA Heroine cichlids are examined in more explicit evolutionary and ecological detail.

## Methods

### DNA Sequencing

Gene sequence for the cytochrome *b *gene (1137 bp) for 17 Heroine species (see Additional file 1) were combined with sequence for numerous species previously analyzed [43,44]. All species sequenced in this study were collected from the wild except for *Archocentrus spinossimus *that was purchased commercially. Collection localities are available in Table 1. All new sequences were submitted to Genbank [Genbank: HM193438-HM193454]. Because we were interested in efficiently resolving a bifurcating topology and estimating branch lengths only one complete sequence per currently named species was utilized in our analyses.

For sequencing, total genomic DNA was isolated from axial muscle using Puregene^© ^extraction. A 1 μl aliquot of this solution was used to provide a DNA template for polymerase chain reaction (PCR). The entire cytochrome *b *gene was PCR amplified using primers in Hulsey *et al*. [43]. Amplifications were carried out using standardized PCR protocols. The PCR volume was 25 ml (18 ml H2O, 2.75 ml MgCl2 PCR buffer, 1.25 ml MgCl2, 2.0 ml dNTPs (10 mM), 1.25 ml of each primer (10 mM), 0.25 ml Taq and 0.5 ml DNA; approx. 15-20 ng). Thermal cycling conditions consisted of an initial denaturation step of 94°C (2.0 min), 54°C (1.0 min) and 72°C (1.5 min). A final incubation of 72°C for 4 min was added to ensure complete extension of amplified products. Subsequently, the 1.4 kb PCR products were separated from unincorporated primers and dNTPs using electrophoresis in agarose gels run in Tris-acetate buffer (pH 7.8). Ethidium bromide (1.5 mg/ml) was added to the gels for visualization. Positively amplified DNA was then purified using an enzymatic combination of 1 μl of Exonuclease I and 1 μl shrimp alkaline phosphatase per 10 μl of PCR product. Complete gene sequences were assembled from individual sequencing reactions using the program Sequencher version 4.1 (Gene Codes, Ann Arbor, MI).

### Phylogeny Reconstruction

A combined total of 111 species' sequences were examined in the phylogenetic analysis (Additional file 1). Prior to the analyses, it was unclear exactly which species should be included in the CA Heroine clade and therefore a large number of potential outgroups were included. The putative outgroups utilized that are all from South America were *Acarichthys heckelii*, *Aequidens coeruleopunctatus*, *Cichla monoculus, Cleithracara maronii, Heros appendiculatus, Hoplarchus psittacus, Hypselecara coryphaenoides, Mesonauta festivus, M. insignis, Pterophyllum scalare, Symphysodon aequifasciata*, and *Uaru amphiacanthoides*. Their Genbank accession numbers are included (Additional file 1). For the Bayesian analyses, sequences were aligned using Clustal X [67] and codon positions were defined using MacClade 4.0 [68]. ModelTest 3.06 [69] was used to identify the best model of molecular evolution for each codon site. A model of GTR + I + gamma was found to be the best model for all three positions. The Bayesian analyses were executed to find approximations of the maximum likelihood tree using MrBayes 3.0 [70]. The analyses treated the transition-transversion matrices, number of invariant sites, and gamma shape parameters as unlinked or independent for each codon site. Flat prior probability distribution for all parameters were assumed before analysis. We ran three separate Bayesian analyses for 5,000,000 generations with four Markov chains in each run. We sampled trees from the Markov Chain Monte Carlo (MCMC) search algorithm every 1000 generations. At the end of each analysis, the log-likelihood scores were plotted against generation time to identify the point at which log likelihood values reached a stable equilibrium. In all three, the equilibrium appeared to be reached at approximately 100,000 generations, and therefore, sample points prior to generation 100,000 in each run were discarded as "burn-in" samples. The remaining samples from all runs combined were used to produce a majority rule consensus tree in PAUP* 4.0b10 [71]. The percentage of trees that recovered a particular clade (the clade's posterior probability) was depicted on the single best likelihood tree topology found during the Bayesian analyses.

### Chronogram

To generate a time-calibrated chronogram of CA Heroine diversification, we utilized penalized-likelihood implemented in the program r8s [72]. Optimal smoothing parameters that compensate for rate heterogeneity were estimated using cross validation implemented in r8s. Using the optimal rate smoothing parameter of 0.001 derived from the cross-validation, r8s generated a time-calibrated ultrametric tree that was utilized in the subsequent analyses.

Although it is not necessary to analyze LTT plots using absolute rates of time, we chose to calibrate the topology with what is likely a temporally realistic geologic calibration. To calibrate the topology we used the formation of the Punta del Morro an extension of the Mexican Neovolcanic Plateau to the Gulf of Mexico. This boundary was thought to have formed between 5 and 10 million years ago [73]. We used the "set age" command in r8s to fix the age of the divergence for the two cichlid sister-clades that spanned the formation of this boundary at 7.5 million years, the average of geologic estimates for the Punta del Morro formation. In the Heroine cichlids, only the *Herichthys *species are located north of this region [43]. We therefore used the node separating a representative of these species, *Herichthys cyanoguttatus*, and the species *Vieja fenestrata*, a member of the sister group to *Herichthys*, as the node calibrated by the formation of the Punta del Morro.

### Ancestral Area Reconstruction

To determine the ancestral node in the Heroine phylogeny from which to measure patterns of speciation, we reconstructed the ancestral areas of nodes on the CA Heroine chronogram generated above. All species present in mainland Central America were coded as 1 and all species that occur primarily in South America or in the Greater Antilles were coded as 0. Using the program Mesquite [74], likelihood ancestral state reconstruction of presence in these two areas was estimated. The Markov k-state 1 parameter model (Mk1) was implemented to reconstruct the likelihood of ancestral states at nodes [75]. This type of optimization assumes equal probabilities of transitions and therefore estimates a single parameter for transitions between Central America and other regions. It is important to note that our estimate of the Central American ancestral node is likely biased towards reconstructing a relatively deep node as mostly Central American as there are several hundred South American cichlid outgroups not included in our estimate of the ancestral area. However, most of these outgroups are fairly distantly related to the Central American Heroines, and therefore, they should have limited influence on ancestral area estimation. Ancestral state reconstruction is always sensitive to groups not included in the analysis due to processes such as extinction and exclusion of species [75]. However, the likelihood that this ancestral area would be reconstructed as Central American would never be negligible as the three nodes closer to the present all subtend primarily Central American groups. Furthermore, all phylogenetic studies to date have recovered this group of CA Heroines as largely monophyletic [47]. We depicted the likelihood of the ancestral states we estimated using pie diagrams showing the estimated probabilities that speciation at each reconstructed node on the chronogram took place in either Central America or outside of Central America.

### CA Heroine Lineages Through Time

If CA Heroines underwent an explosive bout of diversification following their colonization of mainland Central America, this pattern should be evidenced by deviations from a constant rate pattern of cladogenesis. Several approaches were used to explore diversification rate variation in the CA Heroine chronogram. First, we used Pybus and Harvey's [35] constant rate (CR) test, which examines the accumulation of lineages through time using the test statistic gamma (γ). Under a pure-birth model of diversification, γ follows a standard normal distribution. Thus, values of γ < -1.645 (the critical value for a one tailed test at α = 0.05) indicate a deviation from a pure-birth model where diversification decreases towards the present as one would expect if CA Heroines underwent a burst of diversification early in their history.

However, the CR test assumes complete taxon sampling. Based on species names available in Fishbase [10] and several recent taxonomic revision [11-13], we included all but 32 of the currently 122 named species in the CA Heroine radiation in our analysis (Additional file 2). To compensate for this incomplete sampling, we generated random topologies for the total number of taxa thought to be extant in the clade and then pruned them to the number of species empirically sampled. We examined γ over a range of expected terminal taxa (i.e., species richness). We used species richness values between 90, which assumes our current tree contains all species, to 122 species, the number current taxonomy suggests is in the clade. This approach provided us with a range of lineage values to examine the sensitivity of our statistical inferences to taxonomy. Our null distributions of γ were obtained using 10000 simulated pure-birth trees with taxon sampling generated with the program Phylogen [76].

When examining variation in diversification rate, incomplete taxon sampling can also increase type I error rates because it effectively results in nodes near the tips of the tree being pruned [77]. Not including the other 32 named species of CA Heroines in our phylogeny could result in erroneously finding an apparent high rate of diversification early in the clade's history. To help alleviate this problem, we also examined the values of the γ statistic as our empirical tree containing 90 species was truncated node by node serially back in time. As the tree was truncated, lineage sampling should become increasingly more complete thereby reducing error in inferences based on the value of γ. To examine the resulting changes in the γ statistic as nodes were truncated, we graphed γ, recalculated for the number of lineages remaining in the phylogeny, versus the time-calibrated estimate of the most recent node removed. As one moved towards the root of the CA Heroine phylogeny, γ would be expected to become more negative if an under-sampling of lineages was obfuscating an early burst of diversification in CA Heroines.

Because there was some ambiguity in our ancestral state reconstructions and relatively low support for some of the deeper nodes in the topology, we also re-examined all of the above analyses in the two major subsections of the CA Heroine clade. This allowed us to determine if our results were heavily contingent upon inferences concerning the deepest nodes. Our results from these analyses of subsets of the phylogeny were consistent with those recovered for the entire topology (results not shown).

If a pure-birth model was the best fit to the lineage accumulation of CA Heroines through time, it would suggest CA Heroines did not experience a burst of diversification when they colonized Central America. By comparing models that incorporate additional rate parameters of lineage accumulation, we determined the best model for the empirically observed pattern of CA Heroine diversification rate variation through time. We fit two candidate rate-constant models, pure-birth (yule process) and birth-death (birth and extinction), and three rate variable models (density dependent exponential, density dependent logistic, and Yule two-rate) to the vector of branching times obtained from the CA Heroine chronogram. These models test several types of alternative patterns of speciation and extinction through time [38]. We calculated the likelihood of each model given the data as implemented in the R-package LASER (version 2.2) [37]. Subsequently, we examined the Akaike Information Criterion (AIC) to evaluate which was the best model given the data. We chose the best model based on the distribution of differences between the AIC scores of all of the rate-constant and rate-variable models using a critical difference in AIC scores of α = 0.05 [57]. These fits were derived from comparisons to 10000 simulated pure-birth trees with taxon sampling generated by the program Phylogen [76].

## Authors' contributions

CDH and JAF conceived of the study. PRH performed most of the sequencing and some of the phylogenetic analyses. CDH and JAH performed and analyzed the LTT analyses. All three authors contributed to the writing of the manuscript.

## Supplementary Material

Additional file 1 **Included cichlid species**. Cichlid cytochrome *b *sequences used to generate the Heroine chronogram. Generic names that are repeated in the list are followed with shortened versions of the genus using multiple letters to clarify the genus to which they are currently assigned. Click here for file

Additional file 2 **Heroine species that were not included in this study**. Putative Heroine species that are extant [10,11,13,51] but are not included in our phylogeny are listed. Click here for file
