# Supplementary material for: Temporal diversification of Central American cichlids
Source: BMC Evol Biol. 2010 Sep 14;10:279. doi: 10.1186/1471-2148-10-279 (PMC2944184; doi:10.1186/1471-2148-10-279)
Supplement: Additional file 1 — Included cichlid species. Cichlid cytochrome b sequences used to generate the Heroine chronogram. Generic names that are repeated in the list are followed with shortened versions of the genus using multiple letters to clarify the genus to which they are currently assigned. [file 1471-2148-10-279-S1.DOC]

Additional file 1. Cichlid cytochrome *b* sequences taken from previous studies and used to generate the Heroine chronogram. Generic names that are repeated in the list are followed with shortened versions of the genus using multiple letters to clarify the genus to which they are currently assigned. For the phylogenetic analyses we included 13 South American non-Heroines used as outrgroups, 12 South American Heroines, 3 Greater Antillean Heroine species, the 67 Central American Heroines listed below, and the 17 species in Table 1.

South American non-Heroines:

*Acarichthys heckelii* [Genbank: DQ990687], *Aequidens coeruleopunctatus* [Genbank: AY843377], *Cichla monoculus* [Genbank: DQ990686], *Cleithracara maronii* [Genbank: AY050614], *Heros appendiculatus* [Genbank: DQ010102], *Heros* sp. [Genbank: DQ990691], *Hoplarchus psittacus* [Genbank: DQ990690], *Hypselecara coryphaenoides* [Genbank: DQ990689], *Mesonauta insignis* [Genbank: DQ990692], *M. festivus* [Genbank: DQ494392], *Pterophyllum scalare* [Genbank: DQ990688], *Symphysodon aequifasciata* [Genbank: DQ990693], *Uaru amphiacanthoides* [Genbank: DQ990694]

South American Heroines:

*Australoheros facetus* [Genbank: AY843387], *Au. scitulus* [Genbank: AY998665], *Au. sp. ‘Jacutinga’* [Genbank: AY998658], *Au. sp ‘Uruguai’* [Genbank: AY998659], *Au. tembe* [Genbank: AY843373], *Caquetaia kraussi* [Genbank: AF009938], *Ca. myersi* [Genbank: AY050615], *Ca. spectabilis* [Genbank: DQ990697], C*ichlasoma atromaculatum* [Genbank: AY843347], *C. festae* [Genbank: AY843352], *C. umbriferum* [Genbank: AY843350]

Greater Antillean Heroines

*Nandopsis haitiensis* [Genbank: DQ494391]), *Na. ramsdeni* [Genbank: AY998668]), *Na. tetracanthus* [Genbank: DQ494386]),

Central American Heroines

*Amatitlania nigrofasciata* [Genbank: DQ990698], *Astatheros alfari* [Genbank: AF009948], *As. altifrons* [Genbank: AY843357], *As. bussingi* [Genbank: AY843391], *As. diquis* [Genbank: AY843358], *As. longimanus* [Genbank: AF009943], *As. macracanthus* [Genbank: AY324018], *As. rhytisma* [Genbank: AY843403], *As. robertsoni* [Genbank: AY323980], *As. rostratus* [Genbank: AF009944], *Amphilophus calobrensis* [Genbank: AY843432], *Am. citrinellus* [Genbank: AY843434], *Am. hogaboomorum* [Genbank: AY843433], *Am. labiatum* [Genbank: U88863], *Am. lyonsi* [Genbank: AY843396], *Am. nourissati* [Genbank: EF436465], *Archocentrus centrarchus* [Genbank: AF009931], *Cichlasoma bocourti* [Genbank: EF436463], *C. grammodes* [Genbank: DQ990718], *C. istlanum* [Genbank: DQ990699], *C. pearsei* [Genbank: DQ494388], *C. urophthalmus* [Genbank: AY050624], , *C. punctatum* [Genbank: AY843361], *C. salvini* [Genbank: AY324027], *C. trimaculatum* [Genbank: AY324030], *Cryptoheros myrnae* [Genbank: AY843360], *Cr. nanoluteus* [Genbank: AY843404], *Cr. sajica* [Genbank: AY843359], *Cr. septemfasciatus* [Genbank: AY843341], *Herichthys bartoni* [Genbank: AY324016], *H. carpintis* [Genbank: AY323997], *H. cyanoguttatus* [Genbank: AY323982], *H. deppi* [Genbank: DQ494384], *H. labridens* [Genbank: AY323998], *H. minckleyi* [Genbank: AY323994], *H. pantostictus* [Genbank: AY323988], *H. steindachneri* [Genbank: AY324012], *H. tamasopoensis* [Genbank: AY324000], *Herotilapia multispinosa* [Genbank: AF009942], *Hypsophrys nicaraguensis* [Genbank: AF009929], *Neetroplus nematopus* [Genbank: AF009928], *Ne. panamensis* [Genbank: AY843435], *Paraneetroplus bulleri* [Genbank: AY324004], *Parachromis dovii* [Genbank: DQ990701], *Parac. loisellei* [Genbank: AY843366], *Petenia splendida* [Genbank: DQ990704], *Rocio octofasciata* [Genbank: AY324017], *Thorichthys callolepis* [Genbank: AY324008], *Tho. ellioti* [Genbank: AY324009], *Tho. helleri* [Genbank: AY324021], *Theraps irregularis* [Genbank: DQ494383], *The. wesseli* [Genbank: AY843384], *The. lentiginosus* [Genbank: DQ494390], *Tomocichla sieboldii* [Genbank: AY843430], *Tomocichla underwoodi* [Genbank: AY843367], *Vieja argentea* [Genbank: DQ494389], *V. breidohri* [Genbank: AY050626], *V. fenestrata* [Genbank: AY324020], *V. godmanni* [Genbank: AY843428], *V. guttulatus* [Genbank: AY324023], *V. heterospilus* [Genbank: AY843414], *V. intermedia* [Genbank: DQ494387], *V. melanura* [Genbank: AY843420], *V. microphthalmus* [Genbank: AY843431], *V. regani* [Genbank: DQ990735], *V. synspila* [Genbank: AY050625], *V. tuyrense* [Genbank: AY843375],
