# Supplementary material for: Temporal diversification of Central American cichlids
Source: BMC Evol Biol. 2010 Sep 14;10:279. doi: 10.1186/1471-2148-10-279 (PMC2944184; doi:10.1186/1471-2148-10-279)
Supplement: Additional file 2 — Heroine species that were not included in this study. Putative Heroine species that are extant [10,11,13,51] but are not included in our phylogeny are listed. [file 1471-2148-10-279-S2.DOC]

Additional file 2. Putative Heroine species that are extant [10, 11, 13, 51] but are not included in our phylogeny. The 32 species are *Amatitlania coatepeque, Ama. kanna, Ama. siquia Amphilophus amarillo, Am. astorquii, Am. chancho, Am. flaveolus, Am. margaritifer, Am. sagittae, Am. xiloaensis,* *Am. zaliosus, Cichlasoma aguadae, C. geddesi, C. ufermanni, C. zebra, C. alborum, C. amarum, C. cienagae, C. conchitae, C. ericymba, C. mayorum, C. stenozonum, C. ornatum , C. troschelii, C. gephyrum, Cryptoheros altoflavus, Paraneetroplus nebuliferus, Rocio ocotal, R. gemmata, Theraps coeruleus, Tomocichla asfraci, Vieja zonata*
